# *Cystoseira spinosa* Polysaccharide: A Promising Natural Source for Antioxidant, Pro-Angiogenic, and Wound Healing Applications: In Silico Study

**DOI:** 10.3390/ph18060774

**Published:** 2025-05-23

**Authors:** Mouhamed Ayad Berfad, Intissar Kammoun, Marwa Lakhrem, Zakaria Boujhoud, Malek Eleroui, Manel Mellouli, Saadia Makni, Majed Kammoun, Riadh Badraoui, Jean Marc Pujo, Hatem Kallel, Ibtissem Ben Amara

**Affiliations:** 1 High Institute of Maine Sciences, Technologies Al-Khoms, Al-Khoms 3400, Libya; mberfad@gmail.com; 2 Laboratory of Medicinal and Environment Chemistry, Higher Institute of Biotechnology, University of Sfax, Sfax 3000, Tunisia; kammoun.intissar0@gmail.com (I.K.); lakhremma@gmail.com (M.L.); aroui.malek@gmail.com (M.E.); majed.kammoun@isbs.usf.tn (M.K.); 3 Laboratory of Health Sciences and Technologies, Higher Institute of Health Sciences of Settat, Settat 26000, Morocco; z.boujhoud@uhp.ac.ma; 4 Laboratory of Anatomopathology, CHU Habib Bourguiba, University of Sfax, Sfax 3029, Tunisia; mellouli.manel@yahoo.fr (M.M.); olfa_saadia@yahoo.fr (S.M.); 5 Department of Biology, University of Ha’il, Ha’il 81451, Saudi Arabia; riadh.badraoui@fmt.utm.tn; 6 Section of Histology-Cytology, Faculty of Medicine of Tunis, University of Tunis El Manar, Tunis 1007, Tunisia; 7 Emergency Department, Cayenne General Hospital, Cayenne 97300, French Guiana; tamac1966@gmail.com; 8 Intensive Care Unit, Cayenne General Hospital, Cayenne 97300, French Guiana; kallelhat@gmail.com; 9 Biome and Immunopathology CNRS UMR-9017, Inserm U 1019, Université de Guyane, Cayenne 97300, French Guiana

**Keywords:** antioxidant activities, *C. spinosa*, polysaccharides, wound healing, computational assay

## Abstract

**Background/Objectives:** This study evaluated the potential of a polysaccharide (PCS) extracted from the brown alga *Cystoseira spinosa* as an antioxidant and anti-inflammatory agent. Collected off the coast of Alkhoms, Libya, PCS was investigated for its wound-healing and pro-angiogenic properties, addressing the need for natural bioactive compounds in therapeutic applications. **Methods:** The monosaccharide composition of PCS was analyzed using HPLC-RID, identifying glucuronic acid and xylose as major components. In vitro tests assessed antioxidant activity, while in vivo experiments on 24 rats evaluated wound healing. Rats were divided into four groups: control (saline), standard drug (CYTOL CENTELLA cream), glycerol, and glycerol+PCS. Wound healing was analyzed macroscopically, histologically, and biochemically. The chick chorioallantoic membrane (CAM) model assessed pro-angiogenic effects, and computational analyses explored COX-2 and VEGF pathways. Pharmacokinetic properties were also evaluated. **Results:** PCS demonstrated significant antioxidant activity and accelerated wound healing after 16 days, with improved wound appearance scores and increased collagen content. Histological analysis confirmed PCS outperformed the standard drug. The CAM model showed PCS increased blood vessel density, length, and junctions while reducing lacunarity. Computational analyses supported involvement of COX-2 and VEGF pathways. Pharmacokinetic assessments indicated good bioavailability, non-inhibition of CYP enzymes, and favorable skin permeability. **Conclusions:** PCS shows promise as a natural bioactive polymer for wound healing and tissue regeneration. Its antioxidant, anti-inflammatory, and pro-angiogenic properties, combined with favorable pharmacokinetics, highlight its therapeutic potential. This study provides new insights into the mechanisms of *C. spinosa* polysaccharides and their application in promoting tissue repair.

## 1. Introduction

Wound healing is a sophisticated and finely regulated biological mechanism consisting of several essential stages: hemostasis, inflammation, cellular proliferation, and tissue remodeling [1]. Various factors can hinder this process, such as an overproduction of free radicals, which damage peripheral cells and increase the risk of microbial infections. When these stages are disrupted, chronic wounds, such as diabetic ulcers, pressure sores, or vascular wounds, may develop. These wounds are often associated with prolonged inflammation, resistance to conventional treatments, and insensitivity of cells to repair signals [2,3].

To optimize healing, scientific research has focused intensively on developing innovative dressings and bioactive compounds capable of supporting tissue regeneration while minimizing scar formation [4]. Among the promising strategies, angiogenesis stimulation—forming new blood vessels—holds a central place [5]. Indeed, adequate vascularization is essential to provide the oxygen and nutrients necessary for the repair of damaged tissues while also reducing excessive inflammation [6]. Furthermore, research actively explores safe and effective pro-angiogenic agents that promote controlled neovascularization. These advances are crucial not only for treating complex wounds but also for the success of regenerative medicine approaches, such as the integration of tissue scaffolds and for managing vascular diseases [7]. Therefore, identifying materials and molecules with stable physicochemical properties and biocompatibility is a major challenge for modern medical science.

Marine algae offer a rich source of bioactive polysaccharides and complex carbohydrates with significant potential for therapeutic applications [8]. These polysaccharides exhibit a diverse array of beneficial properties, including immunomodulatory [9], anti-inflammatory [10], antioxidant [11], antibiotic, antitumor [12] and anticoagulant activities [13]. These polysaccharides’ specific characteristics and bioactivity are closely linked to their unique structure and physicochemical properties, which vary based on the algal species from which they are extracted [8,9,10,11,12,13,14].

These natural macromolecules garner considerable research interest due to their potential in treating various diseases [15]. Beyond their therapeutic potential, these polysaccharides are biodegradable, non-toxic, and possess adhesive qualities, making them well-suited for biomedical applications [14]. An up-and-coming area is their use in skincare, especially for wound healing. Their ability to promote cell regeneration and collagen synthesis and their cost-effectiveness and eco-friendly nature make them an attractive ingredient for skincare products.

Cystoseira is a genus of worldwide distribution, with about 80% of the species occurring along the Mediterranean and adjoining Atlantic coasts [16]. Studies on the phytochemical composition and biological activity of Cystoseira species have revealed their significant biomedical potential. These investigations have mainly focused on the variation in phenolic compounds within the thallus (the main body of the alga) and their nutritional, bioactive, and bioaccessible properties. Analyses have demonstrated that these algae contain substantial bioactive compounds, including phenols, flavonoids, tannins, and saponins [17].

Extracts from *Cystoseira* have exhibited remarkable antioxidant and anti-inflammatory effects [18] and antiproliferative activity against cancer cell lines, notably by inhibiting the growth of HMO2, HepG2, and MCF7 cells [19]. Furthermore, these extracts display antimicrobial [20] and anticoagulant properties [21]. These biological activities are strongly correlated with the content of phenolic and flavonoid compounds, emphasizing the key role of these secondary metabolites in the observed therapeutic effects.

*Cystoseira* species have been extensively studied for their chemical composition; however, the bioactivity of their polysaccharides remains relatively unexplored.

A recent study sought to bridge this gap by investigating polysaccharides extracted from *Cystoseira spinosa*. This research assessed their in vitro antioxidant activity and explored their wound-healing and anti-inflammatory potential. Using a comprehensive approach that combined in vivo, in vitro, and computational analyses, the study evaluated wound-healing efficacy through colorimetric assays, collagen density measurements, and histological examinations. Additionally, it investigated the underlying mechanisms by focusing on key inflammation and tissue repair regulators, specifically cyclooxygenase-2 (COX-2) and vascular endothelial growth factor (VEGF). Computational analyses further provided valuable insights into the pharmacokinetics and bioavailability of these promising polysaccharides, highlighting their potential for therapeutic applications.

## 2. Results and Discussion

### 2.1. Scanning Electron Microscopy of PCS

**Figure 1:**
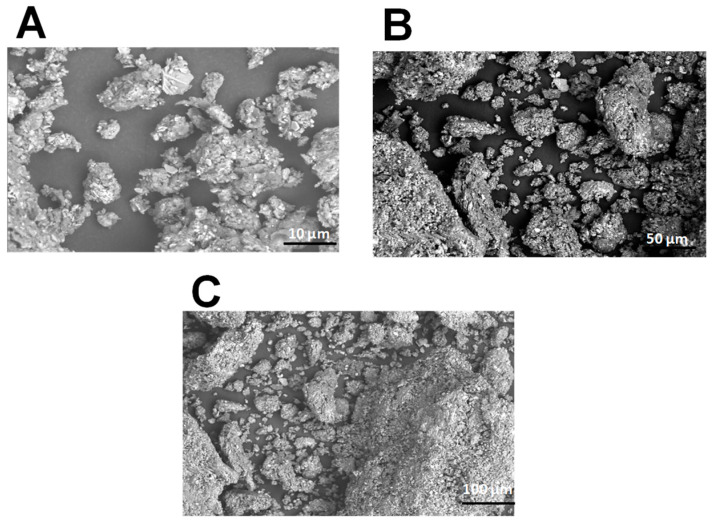
Morphological structure of polysaccharides from *C. spinosa* using scanning electron microscopy in magnifications: (**A**) ×10; (**B**) ×50; and (**C**) ×100.

One method frequently used to ascertain the structural morphology of biopolymers is scanning electron microscopy (SEM). SEM can be used to study the structure of polysaccharides at the nanoscale, which can provide valuable insights into their properties and functions. Figure 1 shows the SEM images of polysaccharides extracted from *C. spinosa*. The pictures reveal the rough surface of PCS at 10, 50, and 100 times magnification.

The results show that the PCS had a rough surface, lumpy, uneven particles, and vast voids that define the porous structure. This aggregation observed in the area of polysaccharides may be attributed to the intense attraction between functional groups on the surface. Polysaccharides from the green alga *Ulva pertusa* had a porous surface comparable to the PCS [22].

### 2.2. X-Ray Diffraction of PCS

**Figure 2:**
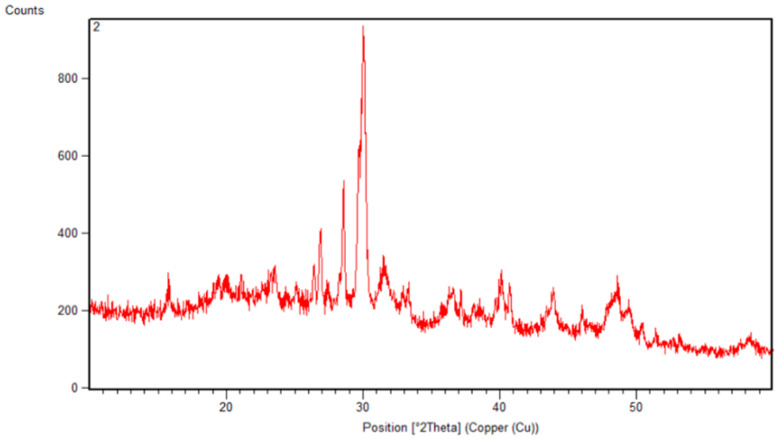
Structure characterization of polysaccharides from *C. spinosa* using X-ray diffraction pattern.

X-ray diffraction is a non-destructive analytical technique that provides information about the crystal structure of materials. To ascertain the crystalline degree and microstructural alterations of the polysaccharide (ranging from 5 to 80°), the XRD pattern was assessed. According to the PCS X-ray diffractogram data presented in Figure 2, the pattern suggests that the structure of the polysaccharide is crystalline. The peaks’ breadth indicates how tiny the crystallites appear to be. The sample seems somewhat crystalline based on the pattern’s overall strength. In line with our results, Gao et al. [22] explained that the sulfated polysaccharides taken out of the alga cell walls had a dense network of crystalline fibers. It is known that the crystalline and semi-crystalline structures of polysaccharides directly impact many properties that depend on the order degree of the material, such as the bulk polymer’s solubility, flexibility, swelling, or opaqueness [22].

### 2.3. Thermogravimetric Analysis

**Figure 3:**
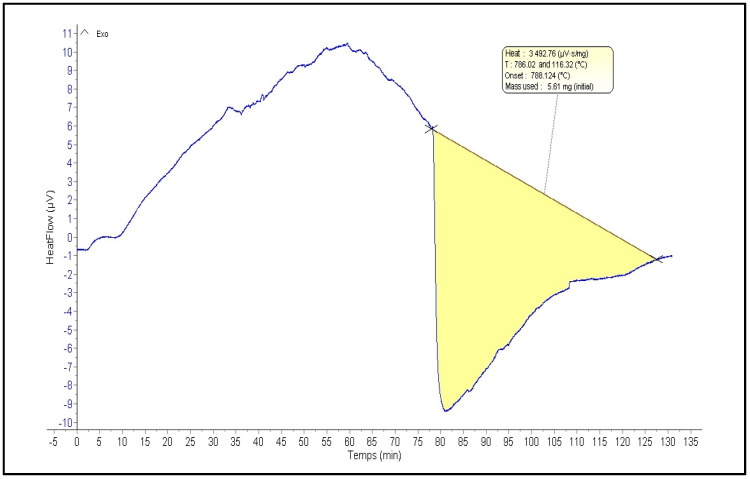
Analysis of the thermal stability of a polysaccharide from *C. spinosa* using *ATG.*

Figure 3 illustrates a thermogravimetric analysis (TGA) curve of a polysaccharide extracted from *C. spinosa*. TGA is a thermal analysis technique that monitors changes in sample mass as a function of temperature and time. Analysis of the TGA curve in Figure 3 reveals a multi-stage decomposition process. The initial stage, observed between 0 and 20 min, involves a minor mass loss of approximately 10%. A more significant mass loss of about 90% occurs in the second stage, from 20 to 80 min. Beyond 80 min, the final stage corresponds to a negligible residual mass. These TGA results provide valuable insights into the polysaccharide’s thermal stability. The polysaccharide exhibits relatively good stability up to approximately 20 min, followed by a rapid degradation phase between 20 and 80 min. The maximum decomposition temperature of the polysaccharide was determined to be approximately 588 °C.

### 2.4. Physical Analysis

Table 1 shows the physical properties of PCS, such as pH, color, and viscosity. The PCS sample exhibited specific color and physical characteristics: a bright appearance (L* = 71.2 ± 0.05), a slight reddish tint (a* = 0.5 ± 0.01), and a subtle yellowish hue (b* = 5 ± 0.05). A 1% PCS solution at 25 °C showed a near-neutral pH of 7.1 ± 0.1. Additionally, the solution’s viscosity was observed to increase proportionally with concentration.

### 2.5. Polysaccharide Composition Analysis by High-Performance Liquid Chromatography-Refractive Index Detector

**Figure 4:**
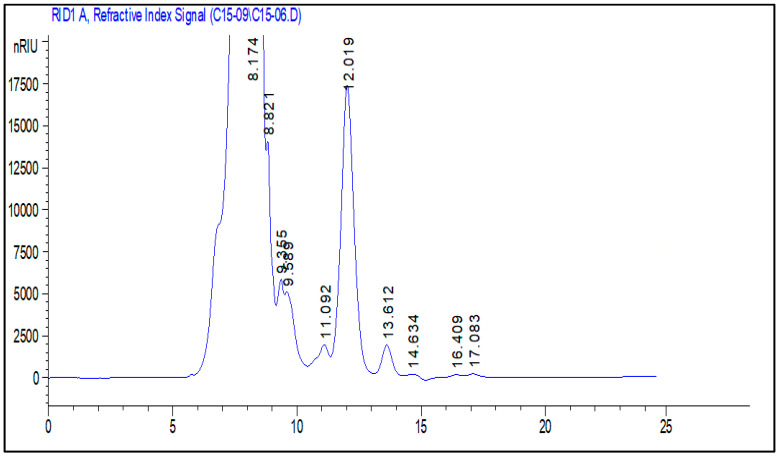
Monosaccharide composition analysis by HPLC-FID of PCS.

High-performance liquid chromatography-Refractive Index Detector (HPLC-RID) was used to analyze the monosaccharide composition of PCS by comparing the retention duration to standards. HPLC-RID analysis of PCS displayed heterogeneous behavior (Figure 4, Table 2), in which glucuronic acid and xylose are the major monosaccharide units at retention times of 8.821 and 12.0191, respectively. In line with our results, the polysaccharide extracted from *Codium bernabei* was composed of arabinose, fucose, xylose, mannose, galactose, and glucose [23]. There are two main sources of this type of diversity in the composition of polysaccharides: endogenous changes in the organisms themselves, such as species type, growth, morphological changes, and reproduction cycle, and exogenous factors like temperature, light day intensity and length, and water’s nutrient concentration [24].

### 2.6. Determination of the In Vitro Antioxidant Properties of PCS

Due to the various ways antioxidants work, evaluating their in vitro antioxidant capability requires a multifaceted approach. This work used a mix of tests, including total antioxidant activity, ion metal chelating activity, and DPPH scavenging activity, to thoroughly and reliably evaluate antioxidant activity.

The free radical scavenging assay tested polysaccharides isolated from *Cystoseira spinosa* for antioxidant properties. One of the most popular and accurate compounds for determining the scavenging capacity of antioxidants is DPPH, a stable free radical with a nitrogen core. Using DPPH as a reagent provides a practical and precise way to titrate the oxidizable groups of natural (or manufactured) antioxidants [25].

**Figure 5:**
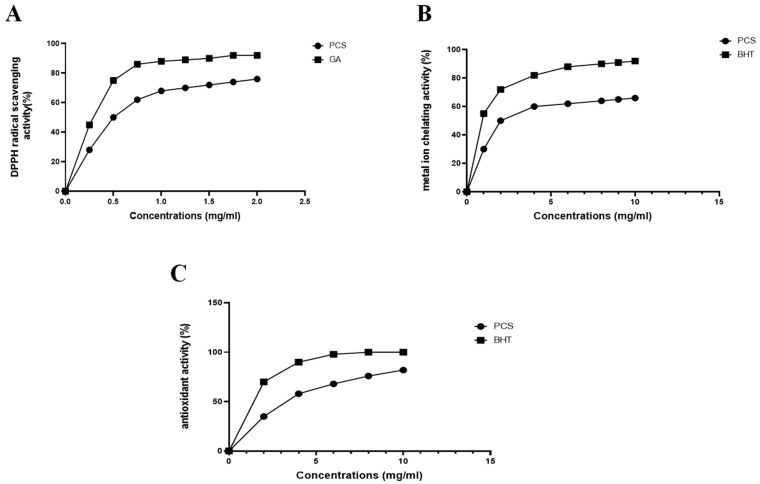
Antioxidant activity of polysaccharide extracted from *C. spinosa* (**A**) DPPH radical scavenging activity, (**B**) ferrous iron chelating activity, and (**C**) total antioxidant activity.

As shown in Figure 5A, the scavenging activity of PCS on a DPPH radical was monitored in a dose-dependent manner. The PCS showed extremely high activity at the final concentration of 2 mg/mL, nearly matching the value of gallic acid. Because of its antiradical solid properties, PCS can halt the chain reaction that initiates the radical process, delaying the commencement of lipid oxidation and potentially offering a health benefit.

Iron, a pro-oxidant, is one of the transition metals most frequently implicated in the spread of the radical chain reaction during lipid oxidation. Chelating drugs have the potential to decrease lipid oxidation by interfering with the formation of the Fe^2+^-ferrozine complex. As evidenced in Figure 5B, the Fe^2+^ chelating activities of PCS and EDTA consistently increased with rising concentrations. PCS demonstrated significant chelating power, achieving an IC50 of 0.2 mg/mL. Specifically, the PCS chelating ratio progressed from 74.9 ± 0.14% at 2 mg/mL to 94.16 ± 2.76% at 4 mg/mL and 98.9 ± 1.54% at 10 mg/mL.

This chelating activity was higher than polysaccharides from the seaweed *Ecklonia cava* (65% at 0.5 mg/mL) [26]. At 5 mg/mL, the Fe^+2^-chelating capacity of PCS was found to be greater than that of polysaccharides derived from fenugreek seeds, recording 42.68% [27].

The phosphomolybdate was utilized generally to estimate the antioxidant capacities of plant extracts. Figure 5C displays the total antioxidant capacity of PCS and BHA, which were used as a reference. The tests were conducted at various doses (0–5 mg/mL). This study’s polysaccharides extracted from *Cystoseira spinosa* show rising antioxidant activity as the concentration increased, reaching its maximal antioxidative efficacy at the highest concentration (5 mg/mL).

Based on the radical termination mechanism, the two main categories of antioxidant capacity measurement techniques are hydrogen atom transfer (HAT) and single electron transfer [14]. The principal antioxidant action of polysaccharides derived from seaweeds is mostly attributed to their ability to scavenge free radicals, such as superoxide, hydroxyl, and 1,1-diphenyl-2-picrylhydrazyl (DPPH) radicals, or to prevent their development [15]; as noted in the same report, sulfated polysaccharide generated and released by marine microalgae has demonstrated the ability to inhibit the formation and activity of reactive chemical species and free radicals. Consequently, the sulfated polysaccharide may function as a defense mechanism against these oxidative and radical stress agents. It has been found that the antioxidant activity of natural polysaccharides is correlated with their molecular weight, water solubility, content of monosaccharides, structure and conformation, polarity, and intramolecular hydrogen bonding [14]. There is evidence to show that the monosaccharide content of polysaccharides has a significant impact on their antioxidant activity. Thus, several studies suggested that polysaccharides containing glucuronic acid, mannose, and arabinose have strong antioxidant properties and might be investigated as a potential source of bioactive chemicals [23]. According to Yarley et al. [8], there is a correlation between the antioxidant activity of polysaccharides and the contents of fructose and glucuronic acid in addition to the molecular weight and sulfated ester concentration. This antioxidant activity is related to the polysaccharides’ ability to chelate metals and scavenge free and hydroxyl radicals. Another experiment revealed that the capacity of polysaccharides to chelate Fe^2+^ was shown to be due to the development of a cross-bridge between COOH in uronic acid and Fe^2+^. Most seaweed-derived polysaccharides have strong antioxidant qualities, potentially benefiting human health by preventing damage caused by reactive oxygen species (ROS) [15].

### 2.7. Evaluation of the Anti-Inflammatory Activity 

Nitric oxide (NO) is a bioactive gas with an unpaired electron where the nitrogen atom is bound to an oxygen atom. It is a byproduct of various processes that occur within the body. iNOS is an inflammation-responsive enzyme expressed by different cell types, including macrophages, responsible for generating NO from arginine and oxygen. Several diseases, including rheumatoid arthritis, osteoarthritis, sepsis, chronic pulmonary inflammatory disease, Crohn’s disease, ulcerative colitis, and carcinogenesis, are brought on by the dysregulation in inflammatory pathways; this hyperactive state leads to the excessive production of a variety of reactive species, including hydrogen peroxide, superoxide anion, hydroxyl radical, and ironically, nitric oxide. This overproduction of reactive species creates oxidative stress, further damaging tissues and perpetuating the inflammatory cycle [28].

**Figure 6:**
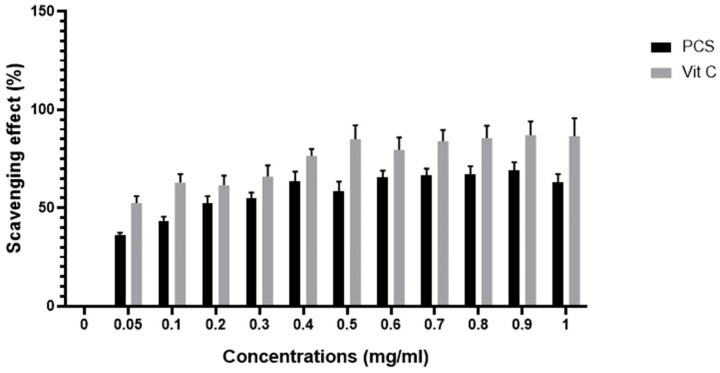
Inhibition curve of nitric oxide radical by polysaccharide from *C. spinosa*; vitamin C was used as a reference.

In our work, the anti-inflammatory activity of PCS was evaluated in vitro by assessing their capacity to suppress the radical NO. The findings in Figure 6 demonstrate PCS’s significantly higher anti-inflammatory activity. The highest inhibiting effect was noted at a dose of 0.8 mg/mL, with an 80% proportion. Several studies show that polysaccharides’ anti-inflammatory properties make them useful in treating inflammatory diseases and acute infections. The observed outcomes align with previous research, notably that of Ananthi et al. [29]. Their study highlighted that water-soluble polysaccharides extracted from the brown alga *Turbinaria ornata* reached peak free radical scavenging efficacy at a concentration of 125 µg/mL, resulting in a 38.8% inhibition rate. Similarly, Ben Saad et al. [30] found that polysaccharides from the red alga *Alsidium corallinum* exhibited maximum NO scavenging activity of 40% at 1 mg/mL

### 2.8. Evaluation of the Pro-Angiogenic Effect of PCS

There has been a great deal of interest in creating novel and efficient treatments for diseases, including cancer and ischemia disorders, which include compromised angiogenesis [31]. Finding and characterizing pro-angiogenic compounds that can promote the formation of new blood vessels—a process known as therapeutic angiogenesis—is one intriguing strategy. Because it provides a practical and trustworthy platform to evaluate the effect of test molecules on blood vessel development, the chick chorioallantoic membrane assay is a commonly used in vivo model for assessing the pro-angiogenic potential of diverse substances [32]. The process of angiogenesis, the formation of new blood vessels from pre-existing ones, is a crucial physiological mechanism that supports tissue growth and repair. In this study, we employed the chick chorioallantoic membrane assay to determine the pro-angiogenic properties of a novel polysaccharide extracted from *C. spinosa.*

**Figure 7:**
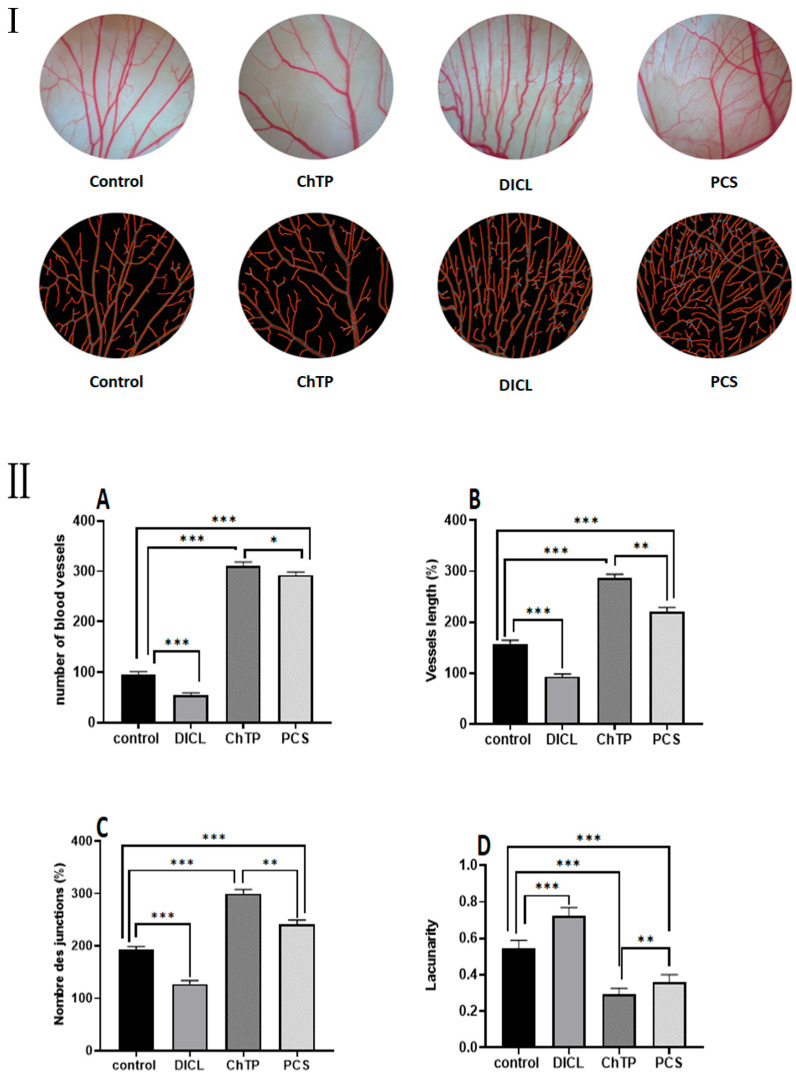
(**I**) The angiogenic activity of PCS using CAM assay. (**II**) (**A**) Number of vessels. (**B**) Length of blood vessels. (**C**) Lacunarity. (**D**) Number of junctions. Values are expressed as means ± SD for 6 eggs in each group. ns: no difference; * *p* < 0.05 ** *p* < 0.01, and *** *p* < 0.001.

This study used six fertilized chicken eggs per group; choriogonadotropin was used as a positive control, and diclofenac was used as a negative control. The influence of PCS on various characteristics, such as the total number of vessels, vessel length, number of junctions, and lacunarity, was determined by image analysis of the CAM membranes, as seen in Figure 7. The distribution of the spaces between the vessels is referred to as lacunarity.

When compared to the control and positive control groups, microscopic analysis showed that PCS significantly increased (*p* < 0.05) blood vessel density, length, and the number of junctions while lowering the lacunarity, or gap between vessels. At 50 µg/g, the PCS induced the most potent effect, similar to the reference medication, choriogonadotropin. In particular, the PCS-treated group showed a decrease in lacunarity (0.36 ± 0.003) and a substantial increase in vessel number (293 ± 4.5), vessel length (220.5 ± 5.5), and junction number (241.25 ± 6.25). Conversely, the control group had the lowest values for these measures, suggesting a vascular network that is sparse and irregular.

The development of new blood vessels, or angiogenesis, is essential to the healing of wounds. It makes removing waste products from metabolism possible while facilitating the flow of nutrients and oxygen necessary for tissue regeneration and cell proliferation. Moreover, angiogenesis promotes the migration of macrophages and fibroblasts, two types of cells involved in tissue healing [6,7,8,9,10,11,12,13,14]. In line with these results, previous research by Cumashi et al. [33] demonstrated that fucoidans from other brown algae, including *Laminaria saccharina*, *Laminaria digitata*, *Fucus serratus*, *Fucus distichus*, and *Fucus evanescens* potently inhibited human umbilical vein endothelial cells (HUVEC) tubulogenesis in vitro. This anti-angiogenic effect was correlated with a significant decrease in plasminogen activator inhibitor-1 (PAI-1) levels in HUVEC supernatants. These findings suggest a potential mechanism for fucoidan-induced inhibition of tubulogenesis, whereby the reduction in PAI-1 activity may promote fibrinolysis and disrupt the formation of stable blood vessels. In this context, PCS, a compound composed of glucose, galactose, and fructose units, directly stimulates ex vivo angiogenesis. This pro-angiogenic activity may be attributed to PCS’s ability to enhance the production of angiogenic growth factors.

### 2.9. In Vivo Experimental Study

#### 2.9.1. Morphological Evaluation

**Figure 8:**
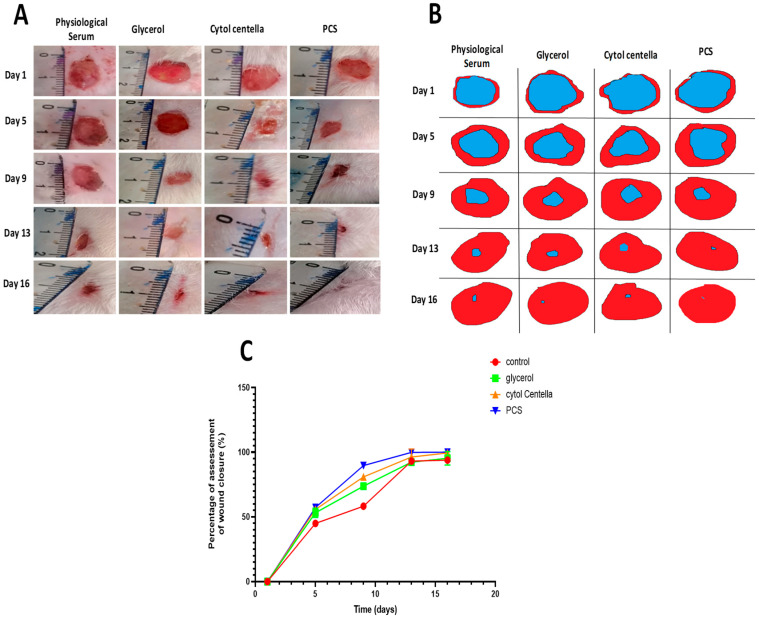
(**A**) Representative photographs of the macroscopic appearance of 1 cm × 1 cm wounds excised on rats at days 1, 5, 9, 13, and 16 of the group treated with physiological serum, “CYTOL CENTELLA”, glycerol and PS + glycerol, (**B**) Traces of wound-bed closure for each group of rats, (**C**) Percentage of wound area contraction of different groups of rats.

Following the circular excision wound model, wound-healing activities were monitored daily for 16 days. The typical photos taken on days 1, 5, 9, 13, and 16 are depicted in Figure 8A,B. All the wounds had a brilliant red hue the day after wound induction, representing the blood covering the underlying muscle. Although the treated groups (PCS and “CYTOL CENTELLA”) had a brown hue, the untreated wounds exhibited a stronger inflammatory side around the wounded skin from the fifth day of therapy.

This hue is associated with scab development, which signals the start of the healing process when blood clots form and the wound’s area noticeably shrinks. For the groups treated with polysaccharide and conventional medication, the area of wounds was reduced, and pinkish-colored tissue was revealed on the ninth day of treatment.

Furthermore, untreated rats still exhibit a dark red, exacerbated inflammation (as measured by glycerol and physiological serum). In the group receiving PCS treatment, full wound closure was seen on the 13th day. This group showed a better appearance of the wound scar, which reflects wound reparations with granulation tissue formation. Nonetheless, the untreated groups continued to exhibit open incisions and scabs. The daily application of PCS with glycerol shows a score equal to 1 after 16 days of treatment; these scores significantly differ from those of the untreated group. The speed of healing is more critical with PCS, which induces almost complete tissue repair. These findings demonstrate PCS’s ability to heal wounds and confirm the possibility that polysaccharides may hasten the healing process. This may be attributed to its significant antioxidant and anti-inflammatory capacity. Similar data were obtained by Sellimi et al. [34], where laminaran purified from *Cystoseira barbata* seaweed demonstrated interesting wound-healing effects.

The size of the region that was wounded each day of the trial was used to measure the wound contraction. The wounds treated with PCS and “CYTOL CENTELLA” had better contraction rates than the control groups (who received glycerol and physiological serum), as seen in Figure 8C. Compared to the other interested groups, rats treated with the polysaccharide and regular medication showed significant healing capability starting on the fifth day. The wounds of the untreated groups remained open on the 16th day of therapy (93.78 ± 0.25%), whereas the groups treated with physiological serum and glycerol healed more slowly than the other groups.

In the meantime, the rats given the polysaccharide treatment had a complete regrowth of the original skin and a 100% reduction in wound size compared to the control group. These results demonstrate that a critical stage in the wound-healing process, wound restoration, is accelerated by our sample; the ability of polysaccharides to stimulate the growth of inflammatory cells and fibroblasts in the wound site and to increase the gap linkage intracellular connection in fibroblast may be the cause of the faster wound contraction. Ayoub et al. [35] showed that laminarin isolated from *Saccharina longicruris* enhanced collagen deposition and produced thicker mesenchymal tissue. This tissue thickness could be increased via secretion and/or deposition of collagen increases. Feki et al. [36] also reported that sulfated polysaccharides extracted from *Falkenbergia rufolanosa* facilitated wound healing in Wistar rats.

#### 2.9.2. Calculating the Amount of Hydroxyproline

**Figure 9:**
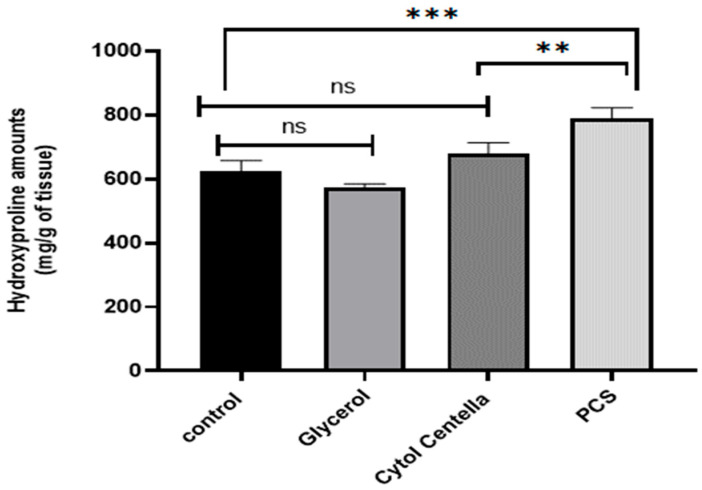
Hydroxyproline concentrations in rat wound tissue biopsies following treatment with *Cystoseira spinosa* polysaccharides. ** *p* < 0.01, and *** *p* < 0.001, ns: no difference.

Hydroxyproline, as a biochemical marker for collagen tissue, is the primary structural protein in the body’s extracellular matrix [37]. The increasing collagen synthesis and deposition displayed a critical role in wound closure [38]. As illustrated in Figure 9, rats treated with PCS (790.275 ± 10.87) and “CYTOL CENTELLA” (677.489 ± 5.43) had greater levels of hydroxyproline (*p* < 0.05) than the control group (623.69 ± 16.31) and the glycerol-treated group (572.39 ± 32.62). Based on these findings, the presence of polysaccharides can stimulate the synthesis of collagen and fibroblasts at the site of the wound, thereby promoting the process of wound healing. The assessment of collagen alteration serves as an indicator of the progress of tissue recovery. Building on this concept, Kumar et al. [39] demonstrated the efficacy of algal polysaccharides in promoting wound closure. These biopolymers possess remarkable mechanical and physical properties and exceptional biocompatibility and biodegradability. These attributes make them prime candidates for regulating coagulation, hemostasis, thrombosis, oxidative stress, protease activity, inflammatory and immune responses, and pain signaling. Consequently, wound healing can be faster by preventing early infection, enhancing blood circulation, and supplying essential nutrients and growth factors to the tissue.

#### 2.9.3. Histological Assessment

**Figure 10:**
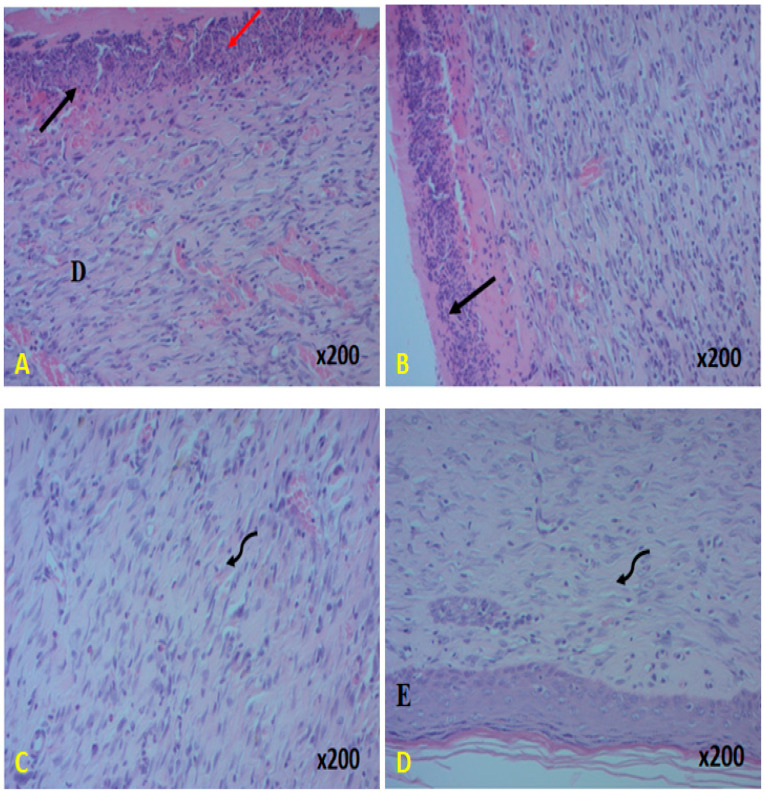
Histological evolution of the effect of the (**A**) physiological serum, (**B**) CYTOL CENTELLA, (**C**) glycerol, and (**D**) PCS + glycerol. Arrows indicate the following: E: Epidermis; D: Dermis. 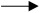 Ulceration 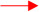 Neutrophil 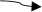 Collagen formation.

The practical technique for assessing the level of wound healing is the histological examination of the injured tissues, as shown in Figure 10. Hematoxylin is used to color the micrograph slices, which are then examined. Light microscopy is used to evaluate the properties of inflammation and regeneration, such as neovascularization, neutrophil infiltration, granulation tissue development, and tissue congestion.

The glycerol-treated and untreated groups show incomplete epithelization, according to the histological assessment of the epidermis (Figure 10). In addition, there was a limited amount of collagen and number of fibroblast forms, necrosis, as well as an invasive inflammatory infiltration seen in the dermis. Nevertheless, better wound reepithelization and neovascularization with a low number of macrophages were seen in the histological observations of the wounds treated with PCS and “CYTOL CENTELLA”, confirming the supply of connective tissue linked to the intriguing elevation and improved collagen and fibroblast fiber organization. This discovery may be connected to the polysaccharide’s antioxidant and anti-inflammatory potential, a key factor in wound healing. Histopathological scores calculated for each group section (Table 3 and Table 4) confirm these data.

Numerous studies have shown that wound healing may result from various processes, including a rise in the rate of neovascularization and re-epithelialization, an increase in the capacity of destructive free radicals, a decrease in inflammation, and the control of infection, which may result from the antioxidant activity of polysaccharide constituents [35,36,40].

Given the circumstances and previously discovered in vitro antioxidant activities, PCS is an antioxidant agent that can effectively squelch free radicals through an electron-donating mechanism, ultimately protecting cells from oxidative damage during wound healing.

### 2.10. Computational Findings

Table 5 shows the pharmacokinetics and medicinal chemistry of *Cystoseira spinosa*-identified monosaccharides based on their ADMET characteristics. The study of ADMET characteristics is important to avoid possible drug failures at advanced drug management stages [41]. Our findings reveal acceptable properties for all the identified monosaccharides. None inhibited the five studied isoforms of cytochrome P450 (1A2, 2C9, 2C19, 2D6, and 3A4). As CYP isoforms have a key role in the metabolism and medicinal processes, their inhibition is usually associated with several drawbacks [41,42]. While *Cystoseira spinosa* monosaccharides are not blood–brain–barrier permeant, they possess good skin permeation and oral bioavailability. Hence, it could be deduced that *Cystoseira spinosa* monosaccharides possess fewer interactions with CYPs and might improve therapeutic safety.

**Figure 11:**
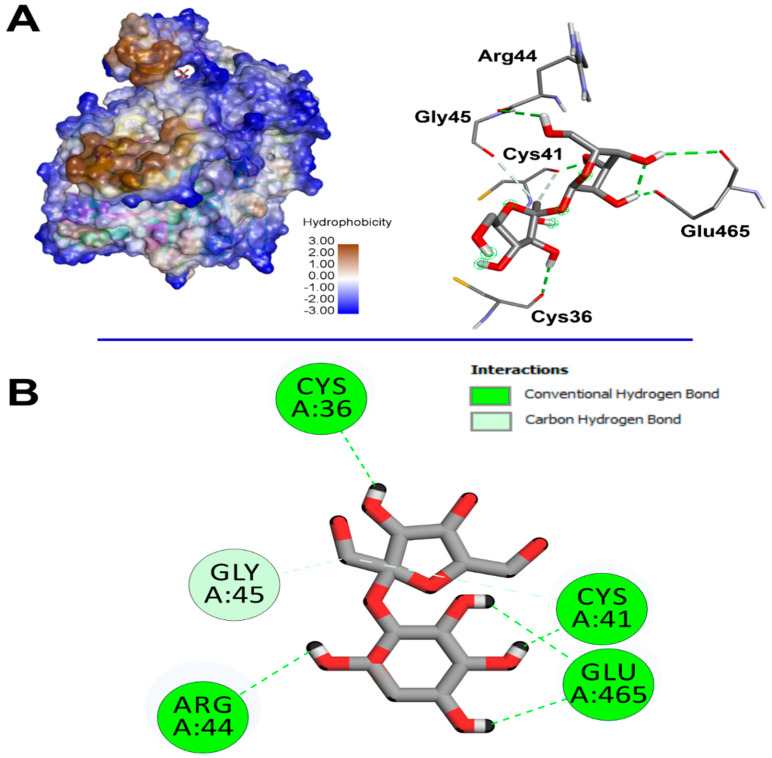
(**A**) 3D illustration of the hydrophobic COX-2 receptor (**left**) and the resulting interactions with the brown algae *Cystoseira spinosa*-derived saccharose (**right**). (**B**) Illustration of the 2D diagram of interactions of saccharose while complexed with COX-2.

**Figure 12:**
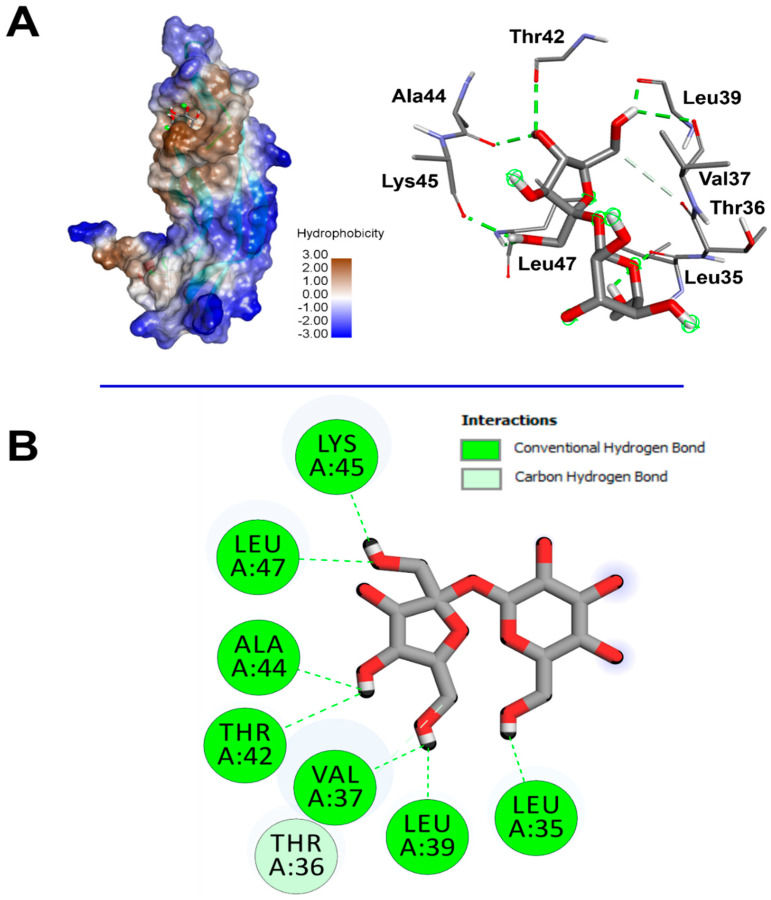
(**A**) 3D illustration of the hydrophobic VEGF receptor (**left**) and the resulting interactions with the brown algae *Cystoseira spinosa*-derived saccharose (**right**). (**B**) Illustration of the 2D diagram of interactions of saccharose while complexed with VEGF.

The computational assay revealed that *Cystoseira spinosa* monosaccharides had different affinities to both COX-2 and VEGF (Table 6). Recent reports linked the binding affinities to the crystal structure and structural geometry of ligands and targeted receptors [29,43]. The binding affinity of *Cystoseira spinosa* monosaccharides reached −7.1 kcal/mol for COX-2 and −5.3 kcal/mol for VEGF, particularly with saccharose. The identified monosaccharides also established good molecular interactions with COX-2 (Table 6, Figure 11) and VEGF (Figure 12). At least four carbon H-bonds were involved in each complex. Our findings also show the implication of Pi-Sigma and carbon H-bonds, which commonly improve the bioactivities and enhance the stability of the complexes [41].

The established molecular interactions involved some key amino acids. For instance, saccharose interacted twice with each of Glu465 and Cys41 and once with each of Cys36, Arg44, and Gly45, once complexed with COX-2. However, it interacted once with each of Leu35, Thr36, Val37, Leu39, Thr42, Ala44, Lys45, and Leu47 while complexed with VEGF. Nevertheless, the highest number of conventional H-bonds was established by rhamnose while complexed with COX-2 as it interacted three times with Thr394, twice with Asn396, and once with each of Gln429 and Glu401. *Cystoseira spinosa* monosaccharides also showed tight embedding on the targeted macromolecules that reached 1.816 Å only. The latter concerned the complex saccharose/COX-2. Recently, it has been reported that short distances (<2.5 Å) between ligands and receptors, similar to those of the current study, are commonly associated with significant bioactivities such as antioxidant, anti-inflammatory, and antimicrobial effects [43,44,45].

Our results confirm the potential therapeutic effect of nature-derived phytochemicals, mainly algae such as *Cystoseira* [29,44].

## 3. Materials and Methods

### 3.1. Chemicals

Absolute ethanol, TFA, 1-1-Diphenyl-2-picryl-hydrazyl (DPPH), ferrozine, sulfuric acid, sodium phosphate, ammonium molybdate, choriogonadotropin, diclofenac, potassium phosphate buffer, potassium ferricyanide, EDTA, and FeCl2, were purchased from Sigma Aldrich (Berlin, Germany).

### 3.2. Sampling and Preparation

**Figure 13:**
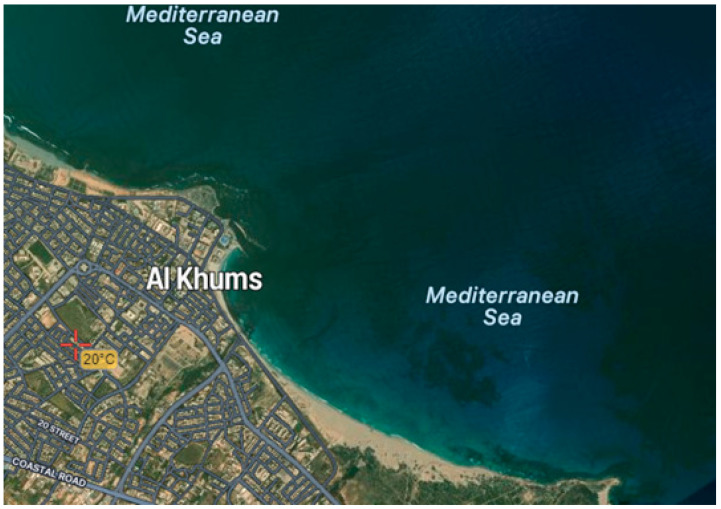
Location of the collection zone (Al-Khums, Libya).

Specimens of *C. spinosa* were collected from the shores of Al-Khums, Libya (Figure 13) in October 2022. The fresh seaweed was meticulously washed with seawater and placed in airtight plastic bags. Upon arrival at the laboratory, the seaweed thalli were thoroughly rinsed with tap water followed by distilled water and subsequently air-dried in a shaded area (25 ± 2 °C) away from direct sunlight and heat for 26 days. The dried seaweed was then pulverized using a mechanical grinder to achieve a homogeneous fine powder (75 µm mesh size). Finally, the powdered seaweed was stored in sealed amber glass bottles at 4 °C for subsequent analysis.

### 3.3. Polysaccharides Extraction

To prepare the polysaccharide used in our study, 70 g of brown algae *C. spinosa* flour were mixed with 1000 mL of distilled water. This mixture was heated to 60 °C for 4 h. The resulting mixture was then filtered under a vacuum to recover the supernatant. Absolute ethanol was added to the supernatant at a volume-to-volume ratio 1:3 to precipitate the sugars. The mixture was then concentrated using a rotavapor. Once concentrated, the recovered mixture was dialyzed against a gradient of distilled water for 48 h. Finally, the sample was dried by lyophilization [46]. The percentage of the extraction yield was calculated as a percentage (%) of the mass (g) of PCS against the initial mass (g) of the algal powder.

### 3.4. Spectroscopic Analysis of PCS

#### 3.4.1. Scanning Electron Microscopy

The microstructure of the PCS was identified by SEM (Termosientific 250 microscope, Hitachi, Tokyo, Japan) operating at 3.0 Kv. The fraction analyzed was photographed with an angle of 90° to the surface.

#### 3.4.2. Monosaccharide Analysis by High Performance Liquid Chromatography-Refractive Index Detector

The monosaccharide composition was determined via HPLC-FID, following the method of Bayar et al. [47]. Briefly, 2 mg of polysaccharide was hydrolyzed in 4 mol/L TFA at 100 °C for 8 h. The resulting hydrolysate (20 µL) was diluted with 980 µL of water and filtered (0.45 µm). HPLC analysis was performed using an Aminex HPX-87H column (Bio-Rad Laboratories, Hercules, CA, USA) at 60 °C, with 0.0005 M H_2_SO_4_ as the mobile phase at a flow rate of 6 mL/min.

#### 3.4.3. X-Ray Diffraction

The XRD pattern of the PCS was obtained using an X-ray diffractometer (D8 advance, Bruker, Berlin, Germany) at room temperature. Data were collected over a 2θ range of 5–80° with a step size of 0.05° and a counting time of 5 s per step.

#### 3.4.4. Thermal Properties of PCS

To evaluate the thermal stability of the PCS, thermogravimetric analysis (TGA) was performed using a Q500 High-Resolution analyzer (TA Instruments, Berlin, Germany) under a nitrogen atmosphere. The PCS powder was heated from 25 °C to 1000 °C at 20 °C per minute.

#### 3.4.5. Physical Analysis

A Color Flex spectro-colorimeter (Hunter Associates Laboratory Inc., Reston, VA, USA) was used to measure the color, with results reported as L* (lightness), a* (redness), and b* (yellowness). The pH was measured using a digital pH meter by fully immersing the glass electrode in a 1% solution. Viscosity was determined using a digital viscometer (NDJ-1, Hitachi, Tokyo, Japan) at 25 °C with a spindle rotation of 30 rpm.

### 3.5. In Vitro Antioxidant Properties of PCS

#### 3.5.1. DPPH Free Radical Scavenging Assay

The 1-1-Diphenyl-2-picryl-hydrazyl (DPPH) technique assessed PCS’s DPPH radical scavenging activity [48]. DPPH• solution (0.2 mmol/L of ethanol) was incubated with varying doses of PCS (0.25–10 mg/mL) for 30 min in the dark. The absorbance was measured at 517 nm using a spectrometer.

The percentage of inhibition (PI) was calculated using the following equation:

PI (%) = (Ac − AS)/AC × 100

AC: the absorbance of the control, AS: the absorbance of the sample. Gallic acid was used as a standard, and all experiments were performed in triplicate.

#### 3.5.2. Ferrous Iron (Fe^2+^) Chelating Activity

The chelating antioxidant activity of PCS on ferrous ions (Fe^+2^) was measured using the Carter method [49]. The Fe^2+^-ferrozine complex’s reduced red hue at 562 nm suggested it could chelate. This formula was used to determine the percentage of inhibition of the formation of the ferrozine-Fe^2+^ complex:

Ferrous ion-chelating activity (%) = [(Ac − As)/Ac] × 100

Ac is the absorbance of the control, and As is the absorbance of the reaction tubes.

#### 3.5.3. Total Antioxidant Activity

Total antioxidant capacity was determined using the phosphomolybdenum assay described by Prieto et al. [50]. This method relies on the sample’s ability to reduce Mo(VI) to Mo(V), leading to the formation of a green phosphate-Mo(V) complex under acidic conditions. The intensity of the green color correlates directly with the sample’s antioxidant activity. Specifically, 500 μL of each sample was mixed with 1 mL of the reagent solution (containing 0.6 M sulfuric acid, 28 mM sodium phosphate, and 4 mM ammonium molybdate). The mixture was then incubated at 95 °C for 90 min in a water bath, and then the mixture was cooled to room temperature. Finally, the absorbance of each sample was measured at 695 nm using a spectrophotometer.

### 3.6. Anti-Inflammatory Activity Assay

The approach revealed by Marcocci et al. [51] was used to calculate the nitric oxide inhibition generated by the PCS. The absorbance was measured at 546 nm and the percentage of NO radical inhibition was calculated below:

% NO inhibition = (A0 − A1)/A0 × 100

A0 and A1 absorbed the blank and polysaccharide solution, respectively.

### 3.7. Angiogenic Potential of PCS

The pro-angiogenic activity of the PCS was assessed using an in-ovo CAM assay, following the methodology of Kohli et al. [52]. Different concentrations of PCB (50 μg/mL) were prepared and sterilized. Choriogonadotropin was used as a positive control. Fertilized chicken eggs were incubated for 3 days; then, 3 mL of albumin was aspirated. On day 9, the chorioallantoic membranes were exposed and treated with different concentrations of PCS. After an additional 3 days of incubation, angiogenesis was evaluated visually and photographed using a stereomicroscope.

### 3.8. Experimental Protocol

#### 3.8.1. Animal

Adult male rats of the Wistar strain weighing approximately 170 ± 220 g were obtained from the Tunisian Central Pharmacy (SIPHAT, ISIN: TN0006670012, Ben Arous, Tunisia). Rats were kept in an air-conditioned room (temperature at 22 ± 3 °C and relative humidity at 40%). They were housed in polycarbonate cages (40/25/20 cm) and provided daily with a standard pellet diet (SNA, Sfax, Tunisia) and water ad libitum. The experimental procedures were carried out according to the “Directive, 2010/63/EU of the European Parliament and of the Council of 22 September 2010, on the protection of animals used for scientific purposes” [53] and were approved by the Higher Institute of Biotechnology (University of Sfax, Tunisia) Ethical Committee (Protocol nº 08.0002/24).

#### 3.8.2. Excision Wound Model

In this technique, the excision model was used to assess wound contraction. Hydrate chloral was used to anesthetize the animals in each group. The rats’ backs had been depilated. Removing the skin made a circular incision of roughly 1 cm by 1 cm (150–200 mm^2^) on each animal’s dorsal side.

#### 3.8.3. Experimental Design

A solution containing 30% glycerol and distilled water was created at 20 mg/mL concentration to make applying the PCS easier. The solution was stirred continuously until a PCS hydrogel formed using the excision model.

After the excision of skin, 24 animals were divided into four groups of 6 animals each:

- Group 1: treated with saline solution (0.9%), and used as a control group;
- Group 2: treated with standard drug “CYTOL CENTELLA” cream;
- Group 3: treated with glycerol;
- Group 4: treated with glycerol + PCS.

Until the wounds were fully healed, the treatments were administered every 2 days. At the end of the experiment, all of the rats were given ether anesthesia, decapitated to end their lives, and their granulation tissues were removed.

#### 3.8.4. Measurement of Wound Area and Contraction Rate

Every wound was checked using digital photography during the treatment, and the area of each wound was physically traced. Autodesk Auto CAD was used to measure the wound surface areas for planning and drafting purposes. The wound contraction rate was calculated according to the following equation:

Rate of wound contraction (%) = (initial surface size − specific day surface size)/initial surface size × 100

The healing activity was evaluated by a method for determining varying scores depending on the size of the wound (Table 7) [54].

On the 16th day, ether was used to anesthetize all the rats, who were then euthanized by decapitation to excise the granulation tissues from them.

#### 3.8.5. Determination of the Content of Hydroxyproline

According to Edwards et al. [55], the hydroxyproline content was measured at 557 nm, and data were reported as mg/g dry weight of tissue.

#### 3.8.6. Histological Examination

Following sacrifice, wound site biopsies were collected from each rat group, fixed in 10% neutral-buffered formalin, and embedded in paraffin. Sections (5 µm) were stained with hematoxylin and eosin (H&E) and examined/photographed using an Olympus CX41 (Berlin, Germeny) light microscope.

### 3.9. Computational Assays

The bioavailability and pharmacokinetic properties of the eight monosaccharides identified in the brown algae *Cystoseira spinosa* were assessed by computational assays as previously described [41,42]. The ADMET (absorption, distribution, metabolism, excretion, and toxicity) properties were also predicted [42,43]. The 3D crystal structure of cyclooxygenase-2 (COX-2, pdb id: 1CX2) and vascular epithelial growth factor (VEGF, pdb id: 2C7W) were retrieved from the RCSB PDB. Both receptors and monosaccharides were prepared and saved in pdbqt format before being subjected to a CHARMm force field as previously described [44,45].

### 3.10. Statistical Analysis

Graph Pad Prism version 9.0, a professional edition, was used to conduct statistical studies utilizing ANOVA analysis at a *p* threshold of 0.05. A 95% confidence level standard deviation was used.

## 4. Conclusions

This research delves into the antioxidant effects and wound-healing capabilities of polysaccharides derived from *Cystoseira spinosa*, a species native to the coast of Alkhoms, Libya. The findings demonstrate that the samples facilitated quicker re-epithelialization and wound closure in Wistar mice subjected to excisional wounds. Furthermore, the polysaccharides exhibited notable antioxidant properties in vitro. These results suggest that polysaccharides from brown algae could offer promising prospects for improving wound-healing treatments. The computational analyses revealed that *Cystoseira spinosa* monosaccharides have strong binding affinities to some key residues of COX-2 and VEGF, which, together with the pharmacokinetic and bioavailability properties, satisfactorily support the experimental findings on antioxidant and wound-healing potentials. Future studies should focus on further elucidating the molecular mechanisms underlying the bioactivity of *Cystoseira spinosa* polysaccharides, particularly their interactions with key cellular pathways involved in wound healing and oxidative stress. Exploring their potential in clinical applications, such as advanced topical formulations or bioactive wound dressings, could pave the way for their translation into effective therapeutic solutions.

## Figures and Tables

**Table 1:** Physical properties of polysaccharide from *C. spinosa.*

Color	PCS
L* (lightness)	71.2 ± 0.05
a* (redness)	0.5 ± 0.01
b* (yellowness)	5 ± 0.05
0.5 g/L	4.6 ± 0.05
1 g/L	6.50 ± 0.45
1.5 g/L	8.5 ± 0.2
pH	7.1 ± 0.1

**Table 2:** Retention time and monosaccharides present in PCS.

RT	Monosaccharide
8.17	Glucuronic acid
9.35	Saccharose
11.09	Glucose
12.01	Xylose
13.61	Fructose
14.63	Galactose
16.40	Arabinose
17.08	Rhamnose

**Table 3:** Histopathologic scores evaluating wounds.

Feature Graded	Grade	Description
Inflammatory infiltrate	1	Profound (>50%)
	2	Scanty (10–50%)
	3	A few (10%)
	4	Absent
Fibroblast proliferation	1	Mild
	2	Moderate
	3	Marked
Collagen formation	1	Mild
	2	Moderate
	3	Marked
New vessels	1	Mild
	2	Moderate
	3	Marked
Epithelium	1	Epithelial necrosis
	2	Epithelial proliferation on the edges of the ulcer
	3	Partial re-epithelialization
	4	Complete re-epithelialization
Epidermal differentiation	1	Basal cells
	2	Spinous epidermal differentiation (early)
	3	Granular epidermal differentiation (late)
	4	Complete

**Table 4:** Histopathological scores on healed wound sections isolated from rats from each group.

Groups	Inflammatory Infiltrate	Fibroblast Proliferation	Collagen Formation	Epithelium	Epidermal Differentiation	Total Score
Control	2	2	3	3	4	14
Glycerol	2	2	2	3	4	13
Cytol Centella	2	3	3	3	4	15
PCS	3	3	3	4	4	17

**Table 5:** Lipophilicity, drug-likeness, pharmacokinetics, and medicinal chemistry based on ADMET (for absorption, distribution, metabolism, excretion, and toxicity) properties of the monosaccharides extracted from *Cystoseira spinosa.*

Entry/Compound Name		1	2	3	4	5	6	7	8
		Arabinose	Fructose	Galactose	Glucose	Glucoronic Acid	Rhamnose	Saccharose	Xylose
Lipophilicity/ Druglikeness	Molecular weight	150.13	180.16	180.16	180.16	397.17	164.16	342.3	150.13
	TPSA (Å^2^)	90.15	110.38	110.38	110.38	114.43	97.99	189.53	90.15
	Consensus Log Po/w	−1.85	−2.04	−2.33	−2.16	0.63	−1.5	−3.29	−2
	Lipinski’s Rule	Yes	Yes	Yes	Yes	Yes	Yes	No	Yes
	Bioavailability Score	0.55	0.55	0.55	0.55	0.55	0.55	0.17	0.55
Pharmacokinetics/ Medicinal Chemistry	GI absorption	Low	Low	Low	Low	High	Low	Low	Low
	BBB permeant	No	No	No	No	No	No	No	No
	P-gp substrate	No	No	Yes	Yes	No	No	Yes	No
	CYP1A2 inhibitor	No	No	No	No	No	No	No	No
	CYP2C19 inhibitor	No	No	No	No	No	No	No	No
	CYP2C9 inhibitor	No	No	No	No	No	No	No	No
	CYP2D6 inhibitor	No	No	No	No	No	No	No	No
	CYP3A4 inhibitor	No	No	No	No	No	No	No	No
	Log Kp (cm/s)	−9.36	−9.42	−9.7	−9.7	−8.3	−8.99	−11.02	−9.36
	Synthetic accessibility	3.8	3.96	4.08	4.08	4.82	3.11	5.16	3.8

**Table 6:** The binding affinity and closest interacting residues of the brown algae *Cystoseira spinosa* identified saccharides with the two targeted receptors: COX-2 and VEGF.

Monosaccharide	Receptor	Binding Affinity (kcal/mol)	Closest Interacting Residues	
			Bond Category/Interacting Residues	Residues (Distance, Å)
Arabinose	COX-2	−5.8	Conventional H-Bonds: Gln461, Cys41, Asn39, Gln461, Cys41, Gly45	Gln461 (2.01)
	VEGF	−4.5	Conventional H-Bonds: Cys61, Cys61, Cys61, Asp64 Carbon H-Bonds: Leu66	Cys61 (2.319)
Fructose	COX-2	−6.4	Conventional H-Bonds: Lys511, Tyr475, Glu520, Tyr475, Glu510	Lys511 (2.089)
	VEGF	−4.5	Conventional H-Bonds: Leu47, Leu74, Leu35, Leu47, Lys45 Carbon H-Bonds: Ala44, Ser50	Leu74 (2.004)
Galactose	COX-2	−6.6	Conventional H-Bonds: Arg120, Ser471, Glu524, Phe470, Glu524, Glu524	Glu524 (2.079)
	VEGF	−4.6	Conventional H-Bonds: Cys61, Cys61, Gly65, Asp64, Leu66, Cys61 Carbon H-Bonds: Asp64, Leu66	Leu66 (1.852)
Glucose	COX-2	−6.3	Conventional H-Bonds: Asn34, Cys47, Ala156, Cys47, Asn39, Cys36, Cys37	Asn34 (2.096)
	VEGF	−4.6	Conventional H-Bonds: Thr42, Val43, Ala44, Val37, Leu39, Lys45, Leu39	Leu39 (2.218)
Glucoronic acid	COX-2	−6.5	Conventional H-Bonds: His207, His207, Thr212, Thr212, Asn382, His388 Carbon H-Bonds: His207, Thr212	His388 (1.973)
	VEGF	−4.4	Conventional H-Bonds: Val37, Gln46, Leu47, Leu47	Leu47 (2.095)
Rhamnose	COX-2	−5.2	Conventional H-Bonds: Thr394, Asn396, Asn396, Gln429, Thr394, Thr394, Glu401 Pi-Sigma: Phe187	Glu401 (2.055)
	VEGF	−4.2	Conventional H-Bonds: Leu47, Lys45, Ala44, Thr42, Ala44, Leu35	Leu47 (1.924)
Saccharose	COX-2	−7.1	Conventional H-Bonds: Cys36, Arg44, Glu465, Cys41, Glu465 Carbon H-Bonds: Cys41, Gly45	Cys41 (1.816)
	VEGF	−5.3	Conventional H-Bonds: Leu47, Val37, Leu39, Thr42, Ala44, Lys45, Leu35 Carbon H-Bonds: Thr36	Leu39 (1.924)
Xylose	COX-2	−6.0	Conventional H-Bonds: Gln461, Glu465, Glu465, Arg44, Gly45	Glu465 (2.010)
	VEGF	−4.1	Conventional H-Bonds: Leu39, Leu35, Thr36, Lys45	Lys45 (2.125)

**Table 7:** Scores for the evolution of experimental burns.

Score	Evaluation of the Healing Process
0	Healing is complete, and tissue repair is complete
1	Tissue healing is almost complete
2	Remains of the crust, the size of the lesion decreases (skin reconstruction)
3	All dead tissue (crusts) are removed, wounds, oozing
4	Necrotic skin is partially removed, ulceration, oozing
5	Necrotic skin completely covers the burned part

## Data Availability

Data is contained in the paper.
